# Acute tryptophan depletion alters affective touch perception

**DOI:** 10.1007/s00213-022-06151-3

**Published:** 2022-05-12

**Authors:** Paula D. Trotter, Sharon A. Smith, David J. Moore, Noreen O’Sullivan, Martyn M. McFarquhar, Francis P. McGlone, Susannah C. Walker

**Affiliations:** 1 Research Centre for Brain and Behaviour, Liverpool John Moores University, Liverpool, UK; 2 Department of Psychology, Liverpool Hope University, Liverpool, UK; 3 Division of Neuroscience and Experimental Psychology, The University of Manchester, Manchester, UK; 4 Institute of Psychology, Health and Society, University of Liverpool, Liverpool, UK

**Keywords:** Touch, Pain, Serotonin, Emotion, Depression, C-tactile afferent, Tryptophan, Social

## Abstract

**Rationale:**

Affiliative tactile interactions help regulate physiological arousal and confer resilience to acute and chronic stress. C-tactile afferents (CTs) are a population of unmyelinated, low threshold mechanosensitive cutaneous nerve fibres which respond optimally to a low force stimulus, moving at between 1 and 10 cm/s. As CT firing frequencies correlate positively with subjective ratings of touch pleasantness, they are hypothesised to form the first stage of encoding affiliative tactile interactions. Serotonin is a key modulator of social responses with known effects on bonding.

**Objectives:**

The aim of the present study was to determine the effect of acutely lowering central serotonin levels on perceptions of CT-targeted affective touch.

**Methods:**

In a double blind, placebo-controlled design, the effect of acute tryptophan depletion (ATD) on 25 female participants’ ratings of directly and vicariously experienced touch was investigated. Psychophysical techniques were used to deliver dynamic tactile stimuli; some velocities were targeted to optimally activate CTs (1–10 cm/s), whereas other, faster and slower strokes fell outside the CT optimal range. Discriminative tactile function, cold pain threshold and tolerance were also measured.

**Results:**

ATD significantly increased pleasantness ratings of both directly and vicariously experienced affective touch, increasing discrimination of the specific hedonic value of CT targeted velocities. While ATD had no effect on either tactile or cold pain thresholds, there was a trend for reduced tolerance to cold pain.

**Conclusions:**

These findings are consistent with previous reports that depletion of central serotonin levels modulates neural and behavioural responsiveness to appetitive sensory signals.

## Introduction

Across the lifespan, supportive relationships enhance health and well-being, while dysfunctional relationships can be precipitating factors in the development of affective disorders (Eisenberger & Cole 2012; House et al. 1988; Walker & McGlone 2013). Touch plays a salient role in close human relationships, supporting healthy development and emotional and social functioning (Burleson & Davis 2013; Dunbar 2010; Gallace & Spence 2010; Oliveras et al. 1990). While a lack of touch in childhood is a significant predictor of adult-onset depression (Brown et al. 2007; Takeuchi et al. 2010), tactile social interactions help regulate physiological arousal and confer resilience to acute and chronic stress (Davidson & McEwen 2012; DeVries et al. 2007; DeVries et al. 2003; Walker 2010).

In recent years, interest has grown in the role a class of unmyelinated, low threshold mechanosensitive cutaneous afferents play in mediating the beneficial effects of tactile social interactions (Morrison et al. 2010; Olausson et al. 2010). Microneurography studies have revealed that the preferred stimulus of these C-tactile afferents (CTs) is a low-force, skin temperature, caress-like stroking touch of between 1 and 10 cm/s (Ackerley et al. 2014; Löken et al. 2009). Psychophysical tests reliably demonstrate participants perceive this same stimulus to be more pleasant than slower or faster velocity touch (Ackerley et al. 2014; Essick et al. 1999; Löken et al. 2009). Their response characteristics led to the hypothesis that CTs encode the affective value of socially relevant tactile interactions (Morrison et al. 2010; Olausson et al. 2010). Indirect support for this *affective touch hypothesis* comes from observational studies where people spontaneously caress their infant or partner at a CT optimal velocity (Croy et al. 2016a; Van Puyvelde et al. 2019). Human behavioural and psychophysiological studies confirm that CT targeted touch has a positive affective value which, through associative learning, can be acquired by affectively neutral stimuli it is experienced with (Pawling, Trotter, McGlone, & Walker, 2017a, b). Furthermore, daily stroking at CT optimal but not faster, non-CT optimal, velocities buffered rats against the behavioural and neuroendocrine effects of exposure to chronic mild stress (Walker et al. 2020).

However, there are individual differences in people’s sensitivity to the specific rewarding value of CT targeted touch (Croy et al. 2016b; Crucianelli et al. 2016; Devine et al. 2020; Krahé et al. 2018; Morrison et al. 2011; Sailer & Ackerley 2017). Adults self-reporting a low frequency of social tactile interactions in daily life rated CT optimal touch as less pleasant than a group who reported frequent social touch (Sailer & Ackerley 2017). A similar blunting of affective touch ratings was observed in a group of young adults who had experienced early life adversity (Devine et al. 2020). Attachment style is also a significant predictor of sensitivity to CT-optimal affective touch (Krahé et al. 2016; Krahé et al. 2018), with securely attached individuals showing greater discrimination in the hedonic value of CT-targeted versus non-CT targeted, faster velocity touch than insecurely attached participants. Loneliness, early life adversity and insecure attachment are all risk factors for the development and maintenance of affective disorders (Kupferberg et al. 2016; McLaughlin & Sheridan 2016; Wang et al. 2018).

Serotonin (5-HT) is a key modulator of social responses with known effects on attachment formation and social bonding (Kiser et al. 2012). Deakin and Graeff (1991) hypothesised the interaction between social stimuli and serotonin is important in the pathogenesis of depression, proposing tactile interactions mediate the protective effects of close personal relationships. Support for this hypothesis comes from rodent studies which show that high levels of maternal licking and grooming confers long-term resilience to stress by increasing 5-HT activity in the hippocampus (Meaney & Szyf 2005). The psychostimulant drug 3,4-methylenedioxymethamphetamine (MDMA) induces rapid 5-HT release and has been labelled an “entactogen” as it increases feelings of closeness to others. MDMA dose-dependently increased ratings of CT optimal but not non-CT optimal touch (Bershad et al. 2019). Though the effect of MDMA on serotonin signalling has been linked to its modulation of social behaviour and may underpin the observed results, MDMA also has indirect effects on the other monoamine transmitters as well as enhancing oxytocin release, so the specific neurobiological basis of these findings is unclear (Dumont et al. 2009; Hysek et al. 2011; Liechti & Vollenweider 2001).

Centrally, serotonin modulates numerous processes underlying mood and reward evaluation through its action on brain regions involved in emotion and cognition (Cools et al. 2008; Kranz et al. 2010). Acute tryptophan depletion (ATD) is a well-established technique for selectively and transiently lowering central 5-HT levels (Bell et al. 2005; Evers et al. 2010; Hood et al. 2005). In a previous fMRI study, we investigated the effect of ATD on neural and behavioural responses to affective touch (Trotter et al. 2016). Neurally, ATD abolished differential responding in the orbitofrontal cortex (OFC) to touch on the arm, which CTs densely innervate, versus the glabrous skin of the fingers, which they do not (though see Watkins et al. 2021). The OFC is important for processing the affective value of sensory inputs with activation in this region often correlating with subjective affective ratings (see Rolls 2019 for recent review). Yet behaviourally, ATD had no differential effect on hedonic ratings of touch in these two locations. However, several previous studies have failed to identify any differences between pleasantness ratings of stroking touch applied on the arm and palm (McGlone et al. 2012; Pawling et al. 2017a) (though see Löken et al. 2011). In contrast, stroking velocity is a reliable predictor of perceived touch pleasantness, with CT targeted touch reliably rated as more pleasant than faster and slower velocities (e.g. Essick et al. 1999). It is currently unclear whether serotonin depletion modulates such hedonic differentiation between CT optimal and non-CT optimal velocities of touch.

Changes in 5-HT transmission modulate responding to both appetitive and aversive stimuli. For example, long-term SSRI treatment, thought to enhance 5-HT activity, is associated with decreased neural responses to both threatening (Harmer et al. 2006) and rewarding stimuli (McCabe et al. 2010). Painful physical symptoms, frequently reported in depression, are remitted by treatment with SSRIs (Wise et al. 2007). Consistently, lowered central serotonin levels, following ATD, have been reported to enhance perception of and reduce tolerance to heat pain (Martin et al. 2017). This finding indicates 5-HT does play a role in modulating affective responses to aversive somatosensory responses. Thus, the aim of the present study was to determine the effect of acutely lowering central serotonin levels on perceptions of appetitive, affective touch.

Using a double blind, placebo-controlled design, we studied the effect of ATD on participants’ ratings of directly and vicariously experienced touch. We used psychophysical techniques to deliver dynamic tactile stimuli; some velocities were targeted to optimally activate CTs (1–10 cm/s), whereas other, faster and slower strokes fell outside the CT optimal range (Löken et al. 2009). To consider top-down effects on these ratings, touch was delivered both using an automated robot and in a social condition by an experimenter (Triscoli et al. 2013). For the vicarious touch ratings, participants viewed a series of short video clips depicting one adult touching another at various upper body sites. We have previously shown that people’s ratings of these stimuli have the same relationship between velocity and anticipated pleasantness as directly felt touch (Devine et al. 2020; Walker et al. 2017a). We hypothesised that, in contrast to the effects of MDMA, ATD would reduce sensitivity to the specific rewarding value of CT-targeted touch. To determine the specificity of any effects observed, we included a standard measure of discriminative touch sensitivity, mechanical detection thresholds. We did not predict any effect of ATD on performance of this task. We also included a test of thermal pain, a cold presser test. Based on previous reports of ATD enhancing perceived intensity and reducing tolerance for heat pain (Martin et al. 2017), we predicted the same effects for cold pain.

## Method

### Participants

Twenty-five healthy female participants aged 18–28 (*M* = 20.92, *SD* = 0.44) were recruited via Liverpool John Moores University. Only female participants were included in this study as they are twice as likely as males to be affected by depression (Hamet & Tremblay 2005) and have been reported to be more susceptible to the effects of the Acute Tryptophan Depletion (ATD) (Bell et al. 2005; Nishizawa et al. 1997). In addition, sex differences have been reported in psychophysical ratings of touch and pain, with women reporting more clinical pain complaints and having lower experimental psychophysical thresholds (Essick et al. 1999; Moore et al. 2013; Smith et al. 2011).

At an initial screening session, a structured clinical interview to diagnose DSM-IV-TR Axis I disorders (SCID) (First et al. 2002) was administered to exclude participants with a history of psychiatric illness. Additionally, participants completed the Beck Depression Inventory (Beck et al. 1961) — a score of less than nine was required to participate. Further inclusion criteria were no history of any neurological disorders, no heart abnormalities or heart conditions, no circulatory problems and normal or corrected to normal vision. All participants were free from chronic pain and reported no pain at the time of testing. Participants were excluded if they were using any medication, except non-steroidal asthma inhalers or hormonal contraceptives, and if they were pregnant. They were also excluded if they had used any street drugs or consumed more than 30 units of alcohol per week or 6 strong cups of tea/coffee per day in the 4-week period prior to testing. During screening, participants were provided with details of the low-protein diet they were to follow the day before each experimental session. They were asked not to eat from midnight onwards on the day of the experimental session, not to drink alcohol for 24 h before each experimental session and not to drink any caffeinated drinks on the morning of each experimental session.

Prior to recruitment, the study was approved by the LJMU research ethics committee (approval reference number: 15/NSP/034). The study complied with the Declaration of Helsinki for Medical Research involving human subjects.

### Materials and measures

#### Tryptophan manipulation

Acute tryptophan depletion (ATD) inhibits serotonin synthesis by reducing the availability of the essential amino acid and serotonin precursor, tryptophan. An amino acid drink devoid of tryptophan is administered, inducing hepatic protein synthesis which depletes circulating tryptophan. Furthermore, the increase in large neutral amino acids competes with the transport of reduced levels of tryptophan across the blood–brain barrier via the large neutral amino acid transporter (Evers et al. 2010; Hood et al. 2005). The control condition is identical except the amino acid drink contains tryptophan. This increases plasma tryptophan, but the ratio of tryptophan to other large neutral amino acids is still reduced, the reduction being significantly greater following ATD (Roiser et al. 2008; Weltzin et al. 1994).

The amino acids were purchased from Nutricia (Liverpool, UK) and Fagron (Rotterdam, the Netherlands). The ratios of amino acids used in the drinks were based on that of Young et al. (1985), but were 80% of the original quantities due to the lower average body weight of females than males (Hood et al. 2005). The amounts used are standard for ATD studies (Bilderbeck et al. 2011; Evers et al. 2006; Trotter et al. 2016). The control drink contained all the amino acids in the quantities listed in Table 1, while the tryptophan depleting drink did not contain the 1.92 g of tryptophan.

**Table 1:** Quantities of amino acids contained in the control drink. The tryptophan depleting drink was the same, except for the omission of l-tryptophan

Amino acid	Quantity (g)
l-Alanine	4.58
l-Arginine	4.08
l-Cystine	2.25
l-Glycine	2.67
l-Histidine	2.67
l-Isoleucine	6.67
l-Leucine	11.25
l-Lysine monohydrochloride	9.17
l-Methionine	2.50
l-Phenylalanine	4.75
l-Proline	10.17
l-Serine	5.75
l-Threonine	5.42
l-Tyrosine	5.75
l-Valine	7.42
l-Tryptophan (TRP + group only)	1.92

The amino acids for each drink, totalling 77.02 g for the control drink and 75.10 g for the tryptophan depleting drink, were weighed out in advance of the experimental session. The drink was made just before consumption on the morning of the testing session. Using a blender, the amino acids were mixed with 150 ml of water and ~ 45 ml of flavouring (chocolate or strawberry ice cream syrup), which is added to make the drink more palatable.

Every participant carried out two experimental sessions on separate laboratory visits, during one session they received the tryptophan depleting drink and during the other session they received the control drink. Drink order delivery was randomised and double blinded. This followed the protocol recommended by Hood et al (2005).

#### Human touch rating task

Participants received manual strokes to the ventral surface of their left forearm, delivered by the experimenter using the middle section of their dominant hand. The length of the participant’s left forearm was measured to find the mid-point, and two dots, 4.5 cm each side of the midpoint, were marked. For consistency of temperature and tactile sensation, the experimenter wore a white cotton glove. The experimenter placed their gloved hand on a 32 °C heat pad for 3 s before touch administration. A visual metronome programmed in PsychoPy2 (https://www.psychopy.org/) was presented on a laptop computer screen placed next to the participant, facing away from them, so that only the experimenter could see the screen. On each of the 9 trials, this guided the researcher in delivering the touch at one of three velocities: static, 3 cm/s and 30 cm/s. Stroking touches were delivered for 6 s; however, due to the unnatural nature of a 6-s static touch (established during pilot testing), static touch was delivered for 3 s. Stroking touches were delivered over 10 cm of the skin in a back-and-forth motion (proximal to distal and distal to proximal). Participants experienced the three velocities three times each in a randomised order. In all conditions, touch was delivered with low force consistent with trying to gently comfort someone.

During each touch trial, participants were shown a computer screen stating the stimulus number, e.g. ‘Stimulus 1’, and provided with the instruction, ‘Please attend to the stimulus’. Once the touch had been delivered, the experimenter pressed the spacebar, which then revealed the rating scale with the question ‘How pleasant was the stimulus?’ above it. The rating scale was a 20 cm long visual analogue scale, with anchors ‘ − 10 Unpleasant’ at the far left and ‘ + 10 Pleasant’ at the far right of the scale. The participant used the left mouse button to rate the perceived pleasantness of the touch they had just experienced. Clicking the left mouse button led to a blue arrow appearing on the screen to visualise their rating. Participants could then alter their rating using the left mouse button if they wished. They then confirmed their rating by pressing the space bar on the keyboard. After they had made their rating, the participant indicated to the experimenter they were ready to progress, and the next trial was initiated. The rating task was programmed in PsychoPy2.

#### Rotary tactile stimulator

Touch was delivered to the volar surface of the left forearm using a rotary tactile stimulator (RTS — Dancer Design, St Helens, UK). The RTS, controlled by a PC running a custom programmed LabView interface, delivers touch stimuli using a rotating probe ‘arm’, with precise force and velocity (Essick et al. 1999; Löken et al. 2009). The RTS was suspended on a stand just above the participant’s arm, and touch was delivered at a force of 0.3 N using a probe with a stroking surface measuring approximately 10 × 2 cm, coated in a soft, smooth, synthetic fabric. On each trial, the RTS delivered a single stroke in a proximal to distal direction at one of 8 velocities (0.2, 0.5, 1, 2, 5, 10, 20 and 50 cm/s). In each session, a participant experienced each velocity 3 times in a randomised order. Software limitations meant the stimuli had to be delivered in two blocks of four velocities. Block A contained the velocities 0.2, 1, 5 and 20 cm/s, and Block B contained the velocities 0.5, 2, 10 and 50 cm/s. Block order was evenly counterbalanced across participants. Participants were asked to wear a blindfold during stimulus application, which they raised to make their rating, then lowered again ready for the next stimulus. Participants rated how pleasant they found the touch on a visual analogue scale ranging from ‘ − 10 Unpleasant’ to ‘ + 10 Pleasant’ which was presented on an iPad.

#### Video rating task

Participants viewed and rated a sequence of 15 short (5 s) video clips (Walker et al. 2017a) presented in a random order depicting one adult actor being touched by another adult actor at 5 different skin sites (4 hairy, back, upper arm, ventral forearm and dorsal forearm; and 1 glabrous, palm) and at 3 different velocities (static, 3 cm/s, 30 cm/s). Immediately after viewing each clip, a new screen appeared where participants were asked to rate, on a Likert scale: How pleasant do you think the action was for the person being touched? 1 (very unpleasant) to 7 (very pleasant).

#### Mechanical touch detection thresholds

Mechanical touch detection thresholds were determined using a standard set of Von Frey filaments (Ugo Basil) which exert forces between 0.008 and 300 g to the skin. Participants were asked to wear a blindfold to ensure that they could not see the probes being applied. A staircase method of limits was used to determine detection thresholds. Starting with a filament which exerted 2 g of force, the filament was applied to the participant’s ventral forearm, and the participants were asked to indicate if they felt any touch. All stimuli were presented with a 1 cm^2^ area of mid-point of the participant’s ventral forearm. For each force, three application windows were presented, in two windows, the stimulus was applied, and one window was used as a catch trial. If the participant felt the sensation in both true trials, then the next finest filament was selected. If this was felt on one trial and not the other, then a third application was used as a tie breaker. Each filament was applied for 2 s with the filament bent. Once the participant reported no longer being able to feel the touch this weight was recorded and the next, higher filament was applied until the participant reported feeling the sensation. This process was repeated for four turnarounds; the threshold and the geometric mean were taken of the 10 values. There was no systematic report of touch detection on catch trials suggesting participants engaged with the task appropriately.

#### Cold pressor test

Cold pressor pain was elicited using a bespoke cold pressor stimulation design (Dancer Design, Liverpool, UK). This consists of a reservoir containing iced water which feeds into a stimulus tank which contains no ice. A thermistor maintains the water at a constant temperature with the water being circulated from the reservoir to prevent a warm pocket forming around the participants’ hand. In the present study, the water was held between 2 and 2.5 °C. Participants were instructed to place their hand into the stimulus tank, without touching the sides and with their fingers splayed. As soon as participant’s hand entered the water a stopwatch was started, they were asked to first indicate when the temperature became painful (pain threshold) and then to remove their hand when they could no longer tolerate the pain (pain tolerance). A maximum duration of 180 s was enforced after which participants were asked to remove their hand from the water.

### Procedure

**Fig. 1:**
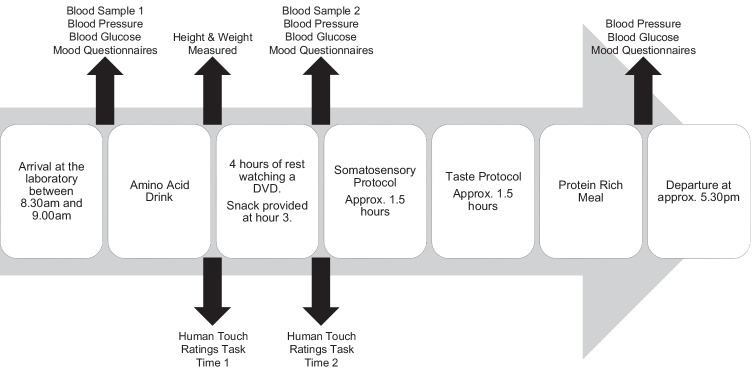
A timeline showing the order of events on a single experimental session. Plasma tryptophan reaches its minimum level 5–6 h after drink consumption (Hood et al. 2005). Therefore, the main somatosensory protocol, reported here, commenced around 4.5 h after drink consumption. The somatosensory protocol comprised the robotic touch ratings, vicarious touch ratings, discriminative touch detection and cold pain perception. The taste protocol, the results of which are reported elsewhere (Smith et al. 2021), was always completed after the somatosensory protocol

Participants were tested individually; they entered the laboratory between 8.30 am and 9 am. They confirmed that they had followed the low protein diet the day before and not eaten since midnight. They then had their blood pressure and blood glucose levels taken. The first of two blood samples were then taken via venepuncture. Following this, they completed the Profile of Mood States (POMS) (McNair et al. 1971) and the Fawcett-Clark Pleasure Scale (FCPS) (Fawcett et al. 1983). Participants were then given the amino acid drink to consume. They were instructed that the entire drink must be consumed within 15 min. Participants then rested for 4 h. During this time, their height and weight were measured, and participants completed a series of questionnaires and the human touch rating task; immediately post-drink and 4 h post-drink. During the rest of the time, participants watched a neutral DVD (David Attenborough’s Blue Planet). The episodes were screened before being shown to participants to ensure the content was neutral. Three hours post-drink participants were given a snack consisting of 4 crackers, 8 g of jam and a pot of fruit jello; the total protein content was < 2 g. Participants drank one pint of blackcurrant cordial, diluted as per bottle instructions (1 part cordial to 4 parts water), just before the second human touch rating task. Approximately, 4 h after drink consumption participants’ blood pressure, blood glucose and mood were measured again (POMS, FCPS and the state version of the State-Trait Anxiety Inventory (STAI-S) (Spielberger et al. 1983) and the second blood sample taken. Approximately, 4.5 h after drink consumption, participants began the experimental phase of the study. They first completed the somatosensory protocol (all of the measures reported here), then a taste perception protocol (reported elsewhere — Smith et al. 2021). At the end of the testing day, at approximately 5 pm, all participants were given a protein-rich meal to replete their endogenous tryptophan levels. Their blood pressure, blood glucose and mood were assessed before they could leave the laboratory. Session 2 took place a minimum of 1 week after session 1. Participants returned to the laboratory, following the low protein diet the day before. The experimental protocol was the same as in session 1, with the exception that the amino acid drink was the one they had not yet consumed. Figure 1 shows the timeline of a single experimental session.

### Data analysis

Total plasma tryptophan was determined by ELISA (Immundiagnostik AG). Data were analysed using SPSS version 25. Differences between the tryptophan depleting and control drink were assessed using a repeated measures ANOVA.

#### Analysis of ratings of directly experienced and vicarious touch

Examination of QQ-normal plots of model residuals revealed the data to be adequately normally distributed. Ratings for directly experienced touch and vicarious touch were analysed using mixed-effects models using the R (R Core Team 2013) lme4 package (Bates et al. 2015). For all models, log_10_ velocity was used, as the velocities included in the study are equidistant on a log_10_ scale. However, as static touch (0 cm/s) cannot be logged, a small constant of 0.1 was added to each velocity before calculating log_10_ velocity when analysing the vicarious and human touch data.

For the human touch data, the dependent variable was the pleasantness ratings, with fixed-effects for the linear and quadratic velocity terms; treatment condition as a factor with two levels, the control and tryptophan depleting amino acid drink; and time as a factor with two levels, baseline vs + 4 h post-drink. A random intercept per participant was included in the model.

For directly experienced robotic touch, delivered using the RTS, the dependent variable was the pleasantness ratings, with fixed-effects for the linear and quadratic velocity terms and treatment condition as a factor with two levels: the control and tryptophan depleting amino acid drink. A random intercept per participant was included in the model.

For vicarious touch responses, the dependent variable was the pleasantness ratings, with fixed-effects for the linear and quadratic velocity terms; touch location as a factor with two levels, hairy and glabrous skin sites; and treatment condition as a factor with two levels, the control and tryptophan depleting amino acid drink. A random intercept per participant was included in the model. Due to the small sample size in this study, to increase power in our analysis, we collapsed over touch location. Since in previous studies with these stimuli, ratings of touch on the palm reliably show a different relationship between pleasantness and velocity than all other skin sites (e.g. Devine et al. 2020; Walker et al. 2017a, b); here, we compare ratings on hairy and glabrous skin.

For all models, omnibus effects were tested using asymptotic type III Wald c^2^ tests using the ANOVA function from the car package (Fox & Weisberg 2019). Significant interactions were followed up using the *testInteractions* function from the phia package (De Rosario-Martinez 2015).

Pain and discriminative touch data was found not to conform to a normal distribution (z-statistic outside of the range − 2.56 and 2.56 (Clark-Carter 2004)) including outlying values (participant values greater than 3SD from the group mean) (Stevens, 1996); thus, non-parametric analyses were conducted to analyse these data. For the discriminative touch task (Von Frey Filaments), geometric means were used to identify punctate touch detection thresholds following ATD and the control drink. Wilcoxon signed rank tests were used to examine whether there were differences in pain threshold and tolerance between the ATD and control session.

## Results

### Plasma tryptophan analysis

Four participants were not included in this analysis due to missing data.

There was a significant interaction between treatment and time (*F*_1,20_ = 150.64, *p* < 0.001, η_*P*_^2^ = 0.88). Analysis of simple main effects identified total plasma tryptophan concentrations significantly decreased 4 h after administration of the tryptophan depleting drink (*F*_1,20_ = 128.721, *p* < 0.001, η_*p*_^2^ = 0.87, *M* = 68.1%, *S.E.* = 0.60%) and significantly increased following the control drink (*F*_1,20_ = 64.75, *p* < 0.001, η_*p*_^2^ = 0.76, *M* = 160.8%, *S.E.* = 4.89%). Total plasma tryptophan concentrations before amino acid drink consumption were comparable (*F*_1, 20_ = 0.297, *p* = 0.59, η_*p*_^2^ = 0.02) but were significantly greater 4 h after administration of the control compared to the tryptophan depleting drink (*F*_1,20_ = 144.34, *p* < 0.001, η_*p*_^2^ = 0.88; see Table 2). Average total plasma tryptophan concentrations reported for this study before and after consumption of the amino acid drinks were similar to those reported in previously published studies using ATD (e.g. Trotter et al. 2016).

**Table 2:** Total plasma tryptophan and mood before and after amino acid drink consumption for both tryptophan depletion and control sessions. Mean values (SD) are presented

	Control		Tryptophan depletion	
	0 h	+ 4 h	0 h	+ 4 h
Plasma TRP (μmol/l)	74.6 (0.66)	185.6 (2.99)	77.9 (0.85)	22.7 (0.32)
POMS total mood	24.13 (7.21)	21.58 (5.94)	24.50 (9.00)	20.96 (4.62)

### Mood

Total scores on the POMS were examined and no significant main effect of Treatment (*F*_1, 23_ = 0.014, *p* = 0.91, η_*p*_^2^ = 0.01), and no significant interaction between treatment and time (*F*_1, 46_ = 0.195, *p* = 0.82, η_*p*_^2^ = 0.008) was identified. Thus, mood was unaffected by the amino acid consumption.

### Human touch ratings

#### Effect of velocity, time (baseline and 4 h post-drink) and treatment on pleasantness ratings

**Fig. 2:**
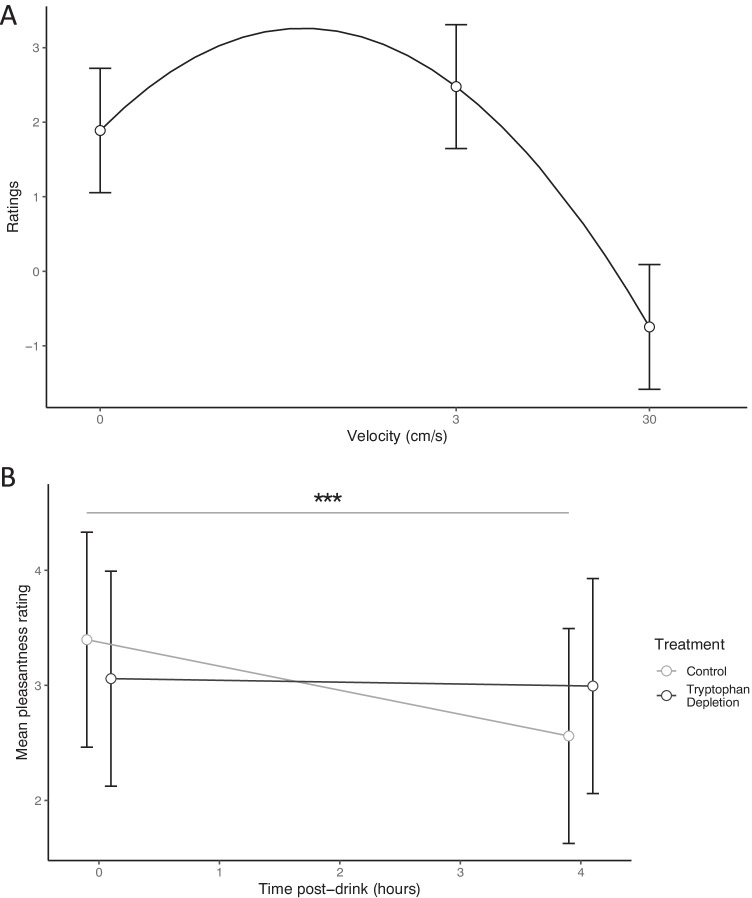
**A** The significant main effect of stimulus velocity, with both linear and quadratic terms accounting for a significant proportion of the variance in ratings (*ps* < 0.001). Mean ratings of 3 cm/s, CT-optimal stroking touch were higher than ratings of static touch and non-CT optimal 30 cm/s strokes. **B** Mean touch ratings (made on a visual analogue scale ranging from ‘10 Unpleasant’ to ‘ + 10 Pleasant’) for experimenter delivered touch in ATD and control conditions at baseline and 4 h post-drink. Note, data points are jittered to increase clarity of error bars. This was significant effect of time (*p* < 0.001), with touch rated significantly more pleasant at baseline compared to 4 h post-treatment. However, while for the control condition, ratings were significantly lower 4 h post-drink compared to baseline (*p* < 0.001***), there was no effect of time on ratings following the tryptophan depleting drink (*p* = 0.244). All error bars =  + / − 95% CIs

The effect of velocity was significant (linear: χ^2^(1) = 181.32, *p* < 0.001; quadratic: χ^2^(1) = 270.91, *p* < 0.001). This reflects the fact that 3 cm/s, CT-optimal stroking touch was rated as more pleasant than either static or non-CT-optimal, 30 cm/s stroking touch. These data are displayed in Fig. 2A.

The effect of time was also significant (baseline vs + 4 h post-drink), (χ^2^(1) = 12.41, *p* < 0.001). This reflects the fact that touch was rated as significantly more pleasant at baseline compared to 4 h post-treatment, regardless of treatment condition.

The main effect of treatment was not significant (χ^2^(1) = 1.17, *p* = 0.280). The interaction of velocity by treatment by time was not significant (linear velocity term × treatment × time: χ^2^(1) = 0.03, *p* = 0.868; quadratic velocity term × treatment × time: χ^2^(1) = 0.43, *p* = 0.512) and neither were the interactions of velocity by treatment (linear velocity term x treatment: χ^2^(1) = 0.58, *p* = 0.445; Quadratic velocity term x treatment: χ^2^(1) = 0.23, *p* = 0.630) or velocity by time (linear velocity term × time: χ^2^(1) = 0.99, *p* = 0.321; quadratic velocity term × time: χ^2^(1) = 0.01, *p* = 0.903).

The interaction of treatment × time was close to significant (χ^2^(1) = 3.52, *p* = 0.061). Further investigation of this interaction identified that, while for the control condition, ratings were significantly lower 4 h post-drink compared to baseline (χ^2^(1) = 14.58, *p* < 0.001); there was no effect of time on ratings following the tryptophan depleting drink (χ^2^(1) = 1.36*, p* = 0.244) (Fig. 2B).

### Robotic touch ratings

#### Effect of velocity and treatment on pleasantness ratings

**Fig. 3:**
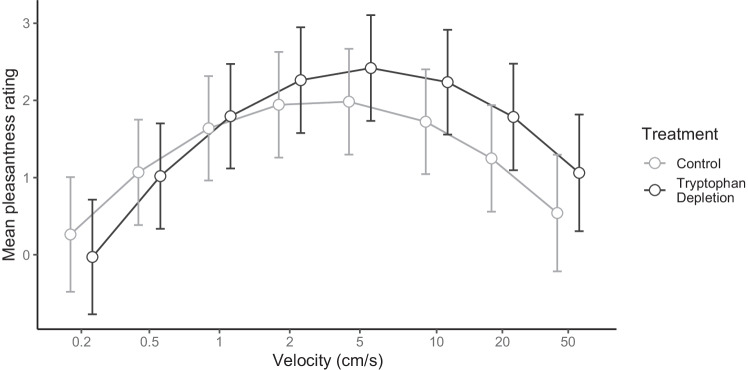
Mean pleasantness ratings (made on a visual analogue scale ranging from ‘10 Unpleasant’ to ‘ + 10 Pleasant’) for RTS delivered touch in the two treatment conditions. Note, for clarity, x-axis labels show the actual velocities applied; however, log_10_ velocity was used in the analysis model and has therefore been plotted. There was a significant main effect of treatment, with ratings significantly higher in the ATD than the control condition (*p* = 0.03). There was a significant main effect of stimulus velocity, with both linear and quadratic terms accounting for a significant proportion of the variance in ratings (*ps* < 0.001). There was also a significant interaction between the linear velocity term and treatment (*p* = 0.020), reflecting a stronger relationship between increasing velocity and increasing touch pleasantness in the tryptophan depletion compared to control condition. Error bars =  + / − 95% CIs. Note, data points are jittered to increase clarity of error bars

The main effect of treatment was significant (χ^2^(1) = 4.70, *p* = 0.030). Pleasantness ratings were significantly increased following tryptophan depletion, compared to the control drink. There was also a significant effect of stimulus velocity. Both the linear and quadratic terms accounted for a significant proportion of the variation in ratings (linear χ^2^(1) = 21.77, *p* < 0.001, quadratic χ^2^(1) = 117.22, *p* < 0.001) (see Fig. 3).

There was also a significant interaction between the linear velocity term and treatment (χ^2^(1) = 5.40, *p* = 0.020). Further investigation identified that the linear velocity term was not significant in the control condition, (χ^2^(1) = 2.74, *p* = 0.098) but was significant in the tryptophan depletion condition (χ^2^(1) = 24.44, *p* < 0.001), meaning there was a stronger relationship between increasing velocity and increasing touch pleasantness in the tryptophan depletion compared to control condition. The interaction of the quadratic velocity term by treatment was not significant, (χ^2^(1) = 0.52, *p* = 0.473).

### Vicarious touch ratings

#### Effect of velocity, skin type (hairy vs glabrous) and treatment on pleasantness ratings

The three-way interactions of velocity by treatment by skin type were not significant (linear velocity term × treatment × skin type: χ^2^(1) = 0.40, *p* = 0.528; quadratic velocity term × treatment × skin type: χ^2^(1) = 1.49, *p* = 0.222).

**Fig. 4:**
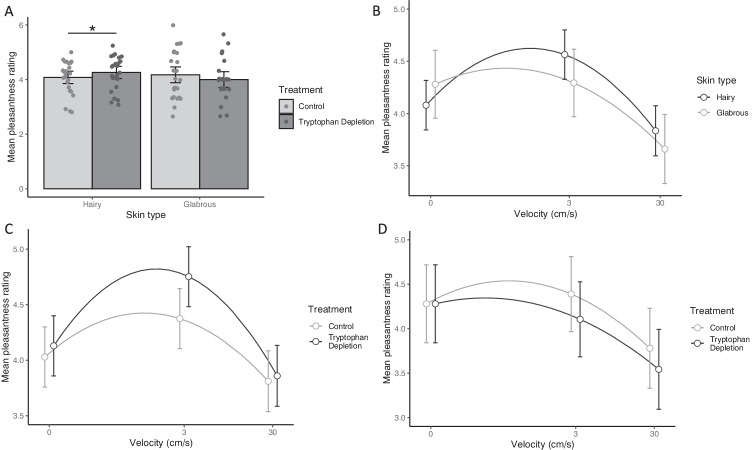
**A** Mean vicarious ratings (made on a 7 point Likert scale where 1 = very unpleasant and 7 = very pleasant) for touch on hairy skin sites compared to the glabrous skin of the palm of the hand in the ATD and control conditions. On hairy skin sites, stimuli were rated significantly more pleasant in the ATD than the control condition (*p* = 0.034*). For touch on the glabrous skin of the palm, there was no significant effect of treatment on pleasantness ratings (*p* = 0.250). **B** Mean vicarious touch ratings at each of the 3 stimulus velocities (static, ~ 3 cm/s and ~ 30 cm/s). The linear velocity term was significantly steeper for the palm than for the other, hairy skin sites (*p* = 0.044). **C** Mean vicarious touch ratings at each of the 3 stimulus velocities (static, ~ 3 cm/s and ~ 30 cm/s) on hairy skin sites in the ATD and control condition. The interaction of the quadratic velocity term by treatment approached significance (χ^2^(1) = 3.78, *p* = 0.052), reflecting the fact the quadratic velocity term, and therefore the inverted-U relationship between velocity and pleasantness, was steeper in the tryptophan depletion condition than in the control condition. **D** Mean vicarious touch ratings at each of the 3 stimulus velocities (static, ~ 3 cm/s and ~ 30 cm/s) on the glabrous skin of the palm in the ATD and control condition. While there was a significant effect of velocity, there was no significant effect of treatment. The interaction between treatment and velocity was not significant (*ps* > 0.5). Note, for clarity in **B**, **C** and **D**, x-axis labels show the actual velocities used; however log_10_ (velocity + 0.1) was used in the analysis model and has therefore been plotted. Also, data points on these figures are jittered to increase clarity of error bars. All Error bars =  + / − 95% CIs

There was a significant treatment by skin type interaction (χ^2^(1) = 4.40, *p* = 0.036). This reflects the fact that, on hairy skin sites, stimuli were rated significantly more pleasant in the tryptophan depletion condition than in the control condition (χ^2^(1) = 5.70, *p* = 0.034), while for the glabrous skin of the palm, there was no significant effect of treatment on pleasantness ratings (χ^2^(1) = 1.32, *p* = 0.250) (see Fig. 4A).

The interactions of velocity by treatment were not significant (linear velocity term × treatment: χ^2^(1) = 0.39, *p* = 0.530; quadratic velocity term × treatment: χ^2^(1) = 0.22, *p* = 0.640). The quadratic velocity term by skin type interaction was not significant (χ^2^(1) = 1.80, *p* = 0.180); however, there was a significant linear velocity term by skin type interaction (χ^2^(1) = 4.04, *p* = 0.044) reflecting the fact the linear velocity term was significant for the glabrous skin of the palm (χ^2^(1) = 8.97, *p* = 0.006), but was not significant for hairy skin sites (χ^2^(1) = 2.22, *p* = 0.136) (see Fig. 4B).

Both the linear velocity term (χ^2^(1) = 11.19, *p* < 0.001) and the quadratic velocity term (χ^2^(1) = 31.08, *p* < 0.001) accounted for a significant proportion of the variance in pleasantness ratings. However, the main effect of treatment was not significant (χ^2^(1) = 0.002, *p* = 0.968) and neither was the main effect of skin type (χ^2^(1) = 0.98, *p* = 0.323).

#### Exploratory analysis of the effect of velocity and treatment for pleasantness ratings: hairy skin sites only

The main effect of treatment was significant (χ^2^(1) = 6.04, *p* = 0.014), reflecting that overall, touch was rated as significantly more pleasant in the tryptophan depletion condition.

The linear velocity term was not significant (χ^2^(1) = 2.36, *p* = 0.125); however, the quadratic velocity term was significant (χ^2^(1) = 63.30, *p* < 0.001). The interaction of the linear velocity term by treatment was not significant (χ^2^(1) < 0.001, *p* = 0.997); however, the interaction of the quadratic velocity term by treatment approached significance (χ^2^(1) = 3.78, *p* = 0.052), reflecting the fact the quadratic velocity term, and therefore the inverted-U relationship between velocity and pleasantness, was steeper in the tryptophan depletion condition than in the control condition (Fig. 4C). The quadratic velocity term was significant in both the control condition (χ^2^(1) = 18.08, *p* < 0.001) and in the tryptophan depletion condition (χ^2^(1) = 49.00, *p* < 0.001).

#### Exploratory analysis of the effect of velocity and treatment for pleasantness ratings on glabrous skin only

Both the linear velocity (χ^2^(1) = 8.01, *p* = 0.005) and the quadratic velocity term (χ^2^(1) = 5.01, *p* = 0.025) were significant. However, here, there was no significant effect of treatment (χ^2^(1) = 1.18, *p* = 0.277) and no significant velocity by treatment interaction (linear velocity term × treatment: χ^2^(1) = 0.44, *p* = 0.506; quadratic velocity term × treatment: χ^2^(1) = 0.16, *p* = 0.691) (Fig. 4D).

### Discriminative touch detection (Von Frey filaments)

Differences between participants’ discriminative touch detection threshold following the ATD and control drinks was assessed using a Wilcoxon-signed rank test. This revealed no significant differences between the conditions (*Z* = 0.886, *p* = 0.376); see Table 3.

**Table 3:** showing mean (SD) mechanical touch, cold pressor pain and cold pain tolerance thresholds.

Test	Control	ATD
Mechanical touch threshold (g)	0.39 (0.25)	0.37 (0.32)
Cold pressor threshold (s)	9.84 (6.26)	9.78 (7.21)
Cold pressor tolerance (s)	65.39 (51.04)	48.82 (45.06)

### Pain perception (cold pressor test)

**Fig. 5:**
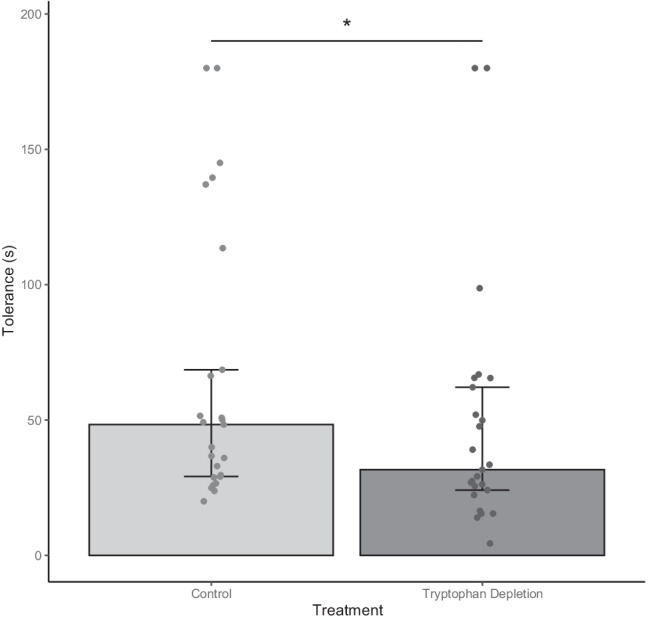
Median tolerance for the cold pain in the ATD and control conditions. Pain tolerance was significantly higher in the control compared to the ATD condition (*p* = 0.049*). Error bars = lower and upper quartiles

Differences between pain threshold and tolerance following the ATD and control drinks were assessed using Wilcoxon-signed rank test (See Table 3). For pain thresholds, this revealed no significant differences between conditions (*Z* = 0.106, *p* = 0.915). However, participants had a significantly higher pain tolerance following the control drink compared to following the ATD drink (*Z* = 1.971, *p* = 0.049). However, this marginal effect needs to be interpreted with caution as one participant kept their hand in the water for the full 180 s in the control condition but took it our immediately in the ATD condition. The effect is no longer significant following removal of this participant (*z* = 1.73, *p* = 0.083) (see Fig. 5).

## Discussion

Our data show that acute depletion of central serotonin levels enhanced hedonic ratings of both directly felt and vicariously experienced affective touch. This was seen in the absence of any change in mood ratings. Overall, ratings of directly felt, robotically delivered touch were increased. For vicariously experienced touch too, we saw that ATD enhanced pleasantness ratings of touch on CT innervated core body sites, and specifically, ratings were enhanced to the CT-optimal (~ 3 cm/s) strokes but not to static or faster (~ 30 cm/s) strokes. We found that with experimenter delivered touch, in the control condition, ratings declined from baseline, at the start of the testing day to 4 h later. In contrast, in the ATD condition, there was no such decline in ratings. These findings fit with previous observations that affective touch ratings can decline over the duration of a lengthy test session (Pawling et al. 2017b). However, here ATD appears to have buffered this effect. Thus, across three different measures, ATD consistently resulted in enhanced ratings of affective touch. These findings are directly contrary to our hypotheses but match those previously reported in response to MDMA, which enhances 5-HT release and blocks reuptake (Bershad et al. 2019). However, taken together with a recent report of intranasal oxytocin enhancing affective touch ratings (Chen et al. 2020), it seems likely the former finding reflects the impact of MDMA on oxytocin rather than 5-HT signalling (Dumont et al. 2009; Kirkpatrick et al. 2014; Walker et al. 2017b).

Notably, our findings were specific to affective aspects of touch as, consistent with our hypothesis, ATD had no impact on discriminative tactile thresholds. While previous studies have reported that central manipulation of 5-HT does modulate somatosensory cortical responses to mechanical touch (Trotter et al. 2016; Waterhouse et al. 1986), detection thresholds which primarily reflect peripheral neural signals have not, to our knowledge, been tested. This suggests that the effects we report on touch reflect a loss of 5-HT modulation of affective, rather than discriminative sensory brain regions, and are consistent with a previous study on taste processing where we found ATD enhanced the perceived intensity and aversiveness of a bitter taste without affecting detection thresholds (Smith et al. 2021).

Though studies of 5-HT function, due to its association with anxiety and depression, frequently focus on processing of threats and punishments (Harmer et al. 2006), changes in 5-HT transmission modulate responding to both appetitive and aversive stimuli. Of particular relevance to the present findings, long-term SSRI treatment, thought to enhance 5-HT activity, has been reported to decrease neural responding to rewarding, as well as aversive, images, smells and tastes (McCabe et al. 2010). Thus, it follows logically that a reduction in 5-HT could enhance neural responding to pleasant as well as aversive stimuli, including pleasant touch. The orbitofrontal cortex receives significant input from the ascending 5-HT system and activation of this region has previously been found to correlate with affective ratings of a range of sensory stimuli including affective touch (Rolls 2019 for recent review). Given here our primary finding was an ATD-induced enhancement of touch pleasantness and we have previously found ATD to modulate affective touch processing in this region (Trotter et al. 2016), it seems likely to be the neural basis of the observed effects. Further work is needed to explore this possibility.

Contrary to our hypothesis, we saw no effect of ATD on cold pain threshold, which contrasts with previous reports of ATD reducing heat pain thresholds (Martin et al. 2017). The fact heat pain thresholds are detected as rapid transitions, reliably moving from no pain to a painful percept with a temperature increase of just 1–2 °C, while cold-pain thresholds show higher inter-subject variability, perhaps in part explains the difference in findings between studies (Essick et al. 2004; Morin & Bushnell 1998). Also, while Martin et al. measured thermal pain sensitivity using a phasic pain model, our study used a tonic model of pain. Participant responses to tonic and phasic pain show limited correlation (Granota et al. 2003), suggesting different underlying mechanisms. We did however see a trend for cold pain tolerance to reduce in the ATD session which, while matching the previous heat pain report, is contrary to a previous study which found no effect of ATD on cold presser threshold or tolerance (Abbott et al. 1992). However, there are several differences between this previous study and ours, most notably that participants immersed their hand in a 37 °C water bath for 2 min before putting their hand into the ice bath, which was maintained at between 0 and 1 °C versus 2–2.5 °C in the present study. Thus, this previous test was more aversive than ours, perhaps masking any effects of ATD. In support of this notion, ATD was found to block the analgesic effects of morphine in the cold-presser test, indicating that as pain threshold and tolerance increased, the effects of ATD on perception were apparent (Abbott et al. 1992). More work is needed to draw firm conclusions on the effect of central 5-HT depletion on pain thresholds and tolerance.

The serotonin system is complex and can influence sensory processing at all levels of the neuroaxis (for review, see Sizemor et al. 2020). With respect to somatosensory processing, 5-HT receptors are found in peripheral as well as central terminals of cutaneous afferents. Tactile allodynia is a side effect of the anti-migraine drug sumatriptan, a 5-HT 1B/D receptor agonist. Though the underlying mechanism is not fully understood, it has been hypothesised to be caused by pre-synaptic inhibition of C-tactile afferents in the dorsal horn (Krämer et al. 2007). A strength of the present study is that ATD exerts its effects via depletion of central 5-HT levels (Crockett et al. 2012) due to the competitive uptake of large neutral amino acids across the blood–brain barrier (Hood et al. 2005), without any impact on peripheral 5-HT function. Thus, though we cannot pinpoint the precise neural basis of the present findings, we can be confident they are specific to the modulatory actions of central 5-HT.

There are several limitations to the present study. It was not viable, due to the complexity of the protocols and time constraints we were working within, to counterbalance order of task presentation. Thus, it is possible that order effects could have influenced the results reported. In the robotic touch rating task, participants did not wear headphones; thus auditory cues from the RTS could theoretically have influenced their ratings, though we believe this would be more of an issue for a discrimination than and affective rating task. In the human touch rating task, we opted to maximise ecological validity over stimulus control; thus, the stimuli were not all matched for duration or contact time on the skin. Finally, a different ratings scale was used for the vicarious touch to the directly felt touch protocols, making direct comparison across tests difficult. However, the consistency of findings across three affective touch measures, in the absence of any effect on discriminative touch thresholds, gives confidence that overall ATD enhanced affective touch ratings.

While the neural basis of CT targeted affective processing has been well characterised (Davidovic et al. 2016; Gordon et al. 2013; Mcglone et al. 2012; Morrison 2016; Olausson et al. 2002), the neurochemical basis has received less attention. Here, we provide the first demonstration that acute central depletion of 5-HT levels enhances ratings of socially relevant touch, whether it is experienced first-hand or vicariously viewed. Future work should investigate the neural basis of the observed effects and potential links between the present finding and previous reports of blunted affective ratings of CT-targeted touch in participants reporting insecure attachment styles or a history of disrupted care.
